# Three-dimensional CBCT analysis of root canal morphology of mandibular premolars using two classification systems

**DOI:** 10.1007/s10266-026-01310-w

**Published:** 2026-01-20

**Authors:** Eda Gursu Sahin, Zuhal Ovuz

**Affiliations:** 1 Faculty of Dentistry, Department of Endodontics, Cankiri Karatekin University, Cankiri, Turkey; 2 Faculty of Dentistry, Department of Maxillofacial Radiology, Cankiri Karatekin University, Cankiri, Turkey

**Keywords:** New classification, Root canal morphology, Vertucci classification, Mandibular premolar

## Abstract

In this study, the root canal morphology, radicular groove (RG) presence, and canal separation level (FCAP) in mandibular premolars of a Turkish population were evaluated using cone-beam computed tomography (CBCT). CBCT images of 3594 mandibular premolars from 402 males and 564 females were analyzed. Root canal morphology was classified using Vertucci’s system and Ahmed’s classification. RG presence and location and FCAP levels were also recorded. The most common configuration in Ahmed’s classification was ^1^TN^1^, followed by ^1^TN^1–2^. Vertucci Configuration I was the most frequent, with Configuration V being the second most common. Some canal configurations showed significant sex-related differences (*p* < 0.01), whereas no age-related differences were found. Bilateral symmetry was generally high. RGs were more common in first premolars and were usually located in the middle third. RG presence was associated with Vertucci Configuration V and with the ^1^TN^1–2^ and ^2^TNB^1^L^1^ configurations. FCAP was most frequently located in the middle third of the root. The root canal morphology of mandibular premolars exhibited a high degree of bilateral symmetry, while complex configurations were associated with the presence of RGs and the level of canal separation (FCAP). These findings have clinical relevance for planning canal shaping and irrigation strategies in endodontic treatment.

## Introductıon

The success of endodontic treatment depends on the accurate identification of root canals, effective disinfection, and three-dimensional hermetic obturationn [1]. In this process, detailed knowledge of both the internal and external anatomy of teeth is essential, as well as possible morphological variations [2]. Even external anatomical features such as radicular grooves (RG) are closely linked to root canal morphology and may directly influence canal cleaning, shaping, and obturation [2].

Mandibular first premolars (M1PMs) typically present with a single root and canal, although 2 to 4 roots and multiple canals or apical foramina have been reported [3]. In contrast, mandibular second premolars (M2PMs) usually exhibit simpler morphology, most often with a single root, single canal, and single apical foramen. These morphological variations are influenced by factors such as ethnicity, sex, age, genetics, and environmental conditions; identifying them is crucial for effective endodontic treatment planning and success [1, 2, 4].

Although root and canal morphology has traditionally been studied using radiographic or sectioning techniques, cone-beam computed tomography (CBCT) has emerged in recent years as one of the most reliable methods for such analyses. In this study, CBCT was selected to evaluate the root and canal morphology of permanent mandibular premolars. Widely adopted in dentistry since the 1990s, CBCT allows detailed assessment of the root canal system because of its noninvasive nature, high-resolution 3-dimensional imaging capability, and relatively low radiation dose[1, 5, 6].

Various classification systems have been developed to describe root canal configurations, including those proposed by Weine, Vertucci, and Versiani and Ordinola-Zapata [4, 7]. However, advances in imaging technologies have revealed more complex anatomical variations that cannot be described by these traditional systems [4]. To address these limitations, Ahmed et al. [4, 8] proposed a new system that integrates tooth numbering with root and canal coding. Ahmed’s classification allows detailed characterization of complex canal systems and provides a more comprehensive and descriptive framework in cases where previous classifications are insufficient.

To illustrate this classification, a canal classified as Configuration V in Vertucci’s system originating from the pulp chamber and dividing into two before reaching the apex can be represented in the Ahmed system as ^1^TN^1−2^, ^2^TN^1^B^1^L^1^ or ^2^TN^1^M^1^D^1^.

To the best of the authors’ knowledge, no study has evaluated the root canal morphology of permanent mandibular first premolars in the Turkish population using both the Vertucci and Ahmed classification systems. Therefore, the aim of this study was to compare the root and canal morphology of permanent mandibular first premolars in a Turkish subpopulation on the basis of the Vertucci and Ahmed classifications and to interpret the findings in a clinical context.

Additionally, the literature reports that accessory canals in mandibular premolars are often associated with the RG region [9]. Therefore, the presence of RG was also assessed in this study. In addition, the “first clear apparent position” (FCAP) was recorded to determine the location of canal bifurcations. Classifying FCAP according to its position along the root-coronal, middle, or apical direction contributes to three-dimensional morphological analysis of the canal system.

The aim of this study was to evaluate the root and canal morphology of permanent mandibular first premolars in a Turkish subpopulation in 3 dimensions using CBCT and to comparatively analyze the findings based on the Vertucci and Ahmed classification systems. Furthermore, this study aimed to determine the FCAP levels of canal bifurcations, assess the presence of RG, and explore potential associations between these 2 morphological features and root canal configurations. By applying this approach, this study contributes to the literature concerning the clinical applicability of different classification systems and the endodontic significance of morphological variations in mandibular premolars.

## Materials and methods

### Inclusion and exclusion criteria

CBCT images of mandibular premolars with fully developed roots from individuals aged 18 years and older were included. Teeth with poor image quality, artifacts, root calcifications, resorptions, periapical pathology, or those that had undergone endodontic treatment or restorations were excluded from the analysis.

### Sample selection and anatomical evaluation

After the inclusion and exclusion criteria were met, 3594 mandibular premolars from 966 CBCT scans were deemed suitable for analysis. Anatomical symmetry was assessed by comparing the right and left mandibular premolars of the same individual.

### CBCT imaging and evaluation procedure: FCAP

CBCT scans were acquired using a Castellini X-Rad1 US TR10 Plus device (Castellini S.p.A., Bologna, Italy) with a tube voltage of 90 kVp, a tube current of 5 mA, and a pulse exposure time of 7 s per scan. Imaging parameters included a voxel size of 0.3 mm and a field of view (FOV) of 10 × 8 mm. The obtained data were evaluated under appropriate lighting conditions on a Lenovo 15.0 510 80 SR monitor. CBCT images in DICOM format were reviewed in axial, sagittal, coronal, and cross-sectional planes using Blue Sky Plan DICOM viewer software (Blue Sky Bio, LLC, Libertyville, IL, USA).

During the evaluation, horizontal, sagittal, and coronal sections of each mandibular premolar were continuously examined from the pulp chamber floor to the apex. The root canal configurations of each tooth were determined on the basis of the Ahmed and Vertucci classification systems. In addition, the “first clear apparent position” (FCAP), the location where the canals first clearly diverge, was recorded to identify canal bifurcation points. FCAP was classified according to its position along the root: coronal, middle, or apical.

On the basis of these evaluations, the three-dimensional morphological characteristics of root canal configurations, FCAP levels, potential canal fusion areas, and the presence of RGs in mandibular premolars were analyzed in detail.

### Calibration

Calibration was performed by an endodontist (EGS) and an oral and maxillofacial radiologist (ZO), each with more than 10 years of clinical experience. To assess both inter- and intraobserver reliability, 30 randomly selected CBCT images were evaluated independently by both observers and reevaluated 10 days later. Discrepancies were resolved through discussion until a consensus was reached. Interobserver agreement was assessed using Cohen’s kappa coefficient and was 0.81.

### Recorded data

The recorded data included patient sex, age (grouped as 18–20, > 20–30, > 30–40, > 40–50, > 50–60, and > 60 years), and tooth number. Root canal configurations were classified according to the Ahmed system and the Vertucci system [8, 10]. The number of roots, their positions in cases of multiple roots, and root canal configurations not included in the Vertucci classification (e.g., trifurcation and C-shaped canals) were also recorded. In addition, the presence of RGs, including their distance from the cementoenamel junction (CEJ), length, and position, was assessed, as well as whether the same root canal configuration was observed bilaterally.

In this study, the CEJ was used as the reference point for the root canal, and the level of canal bifurcation was recorded by dividing the root canal into 3 regions: coronal, middle, and apical.

### Statistical analysis

The data from the scanned CBCT images are recorded in a microsoft excel file. The data were analyzed using RStudio (Version 2025.09.2 + 418) [11] with the R programming language [12]. Data reading, cleaning, and reshaping were performed using the *tidyverse* [13] and *readxl* [14] packages, and Monte Carlo simulations for Fisher’s exact test were implemented via the *coin* package [15].

Associations between categorical variables, such as sex, age range, tooth configuration, and root canal configuration (Vertucci and Ahmed classifications), were analyzed using Fisher’s exact test or Pearson’s Chi-square test, depending on the cell frequency. When more than 20% of the expected frequencies were less than 5, Fisher’s exact test with Monte Carlo simulation (10,000 replicates) was applied. Given that this was a retrospective study including all available CBCT scans that met the eligibility criteria, no a priori sample size calculation was performed. The adequacy of the sample size was instead supported by post hoc evaluation of effect sizes using Cramer’s V coefficient for significant associations. Standardized residual analyses were performed to identify specific categories contributing to significant associations.

Bilateral symmetry in root canal configurations (between contralateral teeth) was assessed using the asymptotic general symmetry test (implemented in the *rcompanion* package [16]) and McNemar’s Chi-square test. In addition, Cohen’s kappa coefficient was calculated using the *irr* package [17] to quantify the degree of agreement between contralateral teeth.

Statistical significance was set at *p* < 0.05. Interpretation of the effect size (V) followed conventional thresholds: small (0.10 ≤ V < 0.30), medium (0.30 ≤ V < 0.50), and large (V ≥ 0.50).

## Results

In this study, mandibular premolars in a local Turkish population were evaluated using CBCT. A total of 3594 mandibular premolars from 402 male and 564 female patients aged 18–81 years were included in the study.

### Ahmed’s classification

In accordance with Ahmed’s new classification system, a total of 3584 mandibular premolars were evaluated (Table 1). The majority of teeth exhibited the ^1^TN^1^ configuration (86.42%). The configurations ^1^TN^1–2^, ^2^TN^1^B^1^L^1^, and ^2^TNB^1^L^1^ were significantly more frequent in mandibular first premolars (M1PMs), whereas the ^1^TN^1^ configuration was more prevalent in mandibular second premolars (M2PMs) (*p* < 0.001, *V* = 0.28). No significant differences were observed for the other canal configurations.

**Table 1:** Distribution of premolar teeth according to Ahmed’s classification

	Left 1st PM	Right 1st PM	Left 2nd PM	Right 2nd PM	Overall
Ahmed’s Classification	*n* [%]	*n* [%]	*n* [%]	*n* [%]	*n* [%]
^1^TN^1−2–1^	6 [0.17]	4 [0.11]	2 [0.06]	2 [0.06]	14 [0.39]
^1^TN^1−2^	126 [3.51]	118 [3.28]	22 [0.61]	12 [0.33]	278 [7.74]
^1^TN^1^	714 [19.87]	722 [20.09]	844 [23.48]	826 [22.98]	3106 [86.42]
^1^TN^2−1^	4 [0.11]	4 [0.11]		2 [0.06]	10 [0.28]
^1^TN^2−2^	2 [0.06]				2 [0.06]
^1^TN^3^	2 [0.06]				2 [0.06]
^2^TN^1^B^1−2^L^1^	2 [0.06]		2 [0.06]		4 [0.11]
^2^TN ^1^B^1^L^1^	66 [1.84]	60 [1.67]	6 [0.17]	10 [0.28]	142 [3.95]
^2^TN B^1^L^1^	10 [0.28]	8 [0.22]			18 [0.5]
^2^TN^1^B^1−2−1^L^1^		2 [0.06]		2 [0.06]	4 [0.11]
^3^TNMB^1^DB^1^L^1^		2 [0.06]			2 [0.06]
^3^TN^1^MB^1^DB^1^L^1^				2 [0.06]	2 [0.06]
C-shaped	2 [0.06]	2 [0.06]	2 [0.06]	4 [0.11]	10 [0.28]
Total	934 [25.99]	922 [25.65]	878 [24.43]	860 [23.93]	3594 [100]

Bilateral assessment revealed no significant asymmetry in either M1PMs (*p* = 0.643) or M2PMs (*p* = 0.08), with similar configurations observed on both sides in both tooth groups. The most frequently observed bilateral configuration was ^1^TN^1^, followed by ^1^TN^1–2^ and ^2^TN^1^B^1^L^1^. These findings indicated that root canal morphology in mandibular premolars generally tended toward bilateral symmetry.

**Fig. 1:**
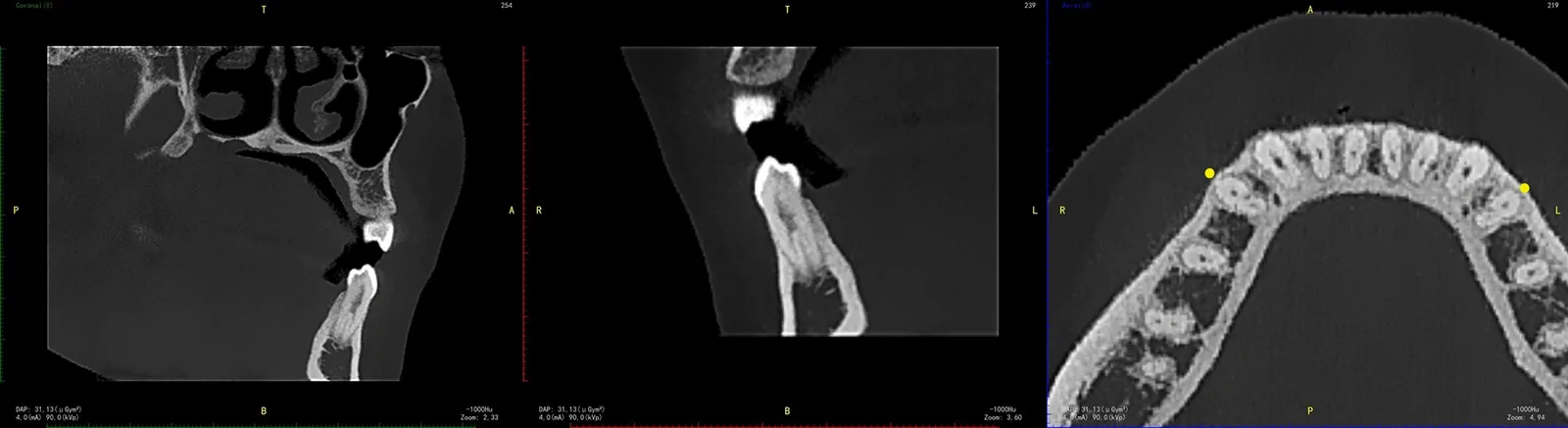
Cross-sectional and axial CBCT images for the ^2^TN^1^B^1^L^1^ configuration

Fisher’s exact test revealed a statistically significant association between canal configuration and age group, although the effect size was weak (*p* < 0.001, *V* = 0.07). The standardized residuals indicated that the ^2^TN^1^B^1^L^1^ configuration (Fig. 1) occurred more frequently than expected in the 18–20 age group and less frequently than expected in the > 50–60 age group, while the distributions of the other configurations were balanced across age groups.

Similarly, a statistically significant association was found between canal configuration and sex, but the effect size remained weak (p < 0.001, V = 0.07). The ^1^TN^1–2^ configuration was more frequently observed in males and less frequently in females, whereas the distributions of the other configurations did not differ significantly by sex. The distribution of canal configurations according to age group and sex is presented in Table 2.

**Table 2:** Distribution of Ahmed’s canal configurations according to sex and age group

	Sex		Age Group						Overall
	Male	Female	18–20	> 20–30	> 30–40	> 40–50	> 50–60	> 60	
Ahmed classification	*n* [%]	*n* [%]	*n* [%]	*n* [%]	*n* [%]	*n* [%]	*n* [%]	*n* [%]	*n* [%]
^1^TN^1−2–1^		14 [0.39]		6 [0.17]	8 [0.22]				14 [0.39]
^1^TN^1−2^	146 [4.06]	132 [3.67]	36 [1]	100 [2.78]	48 [1.34]	62 [1.73]	26 [0.72]	6 [0.17]	278 [7.74]
^1^TN^1^	1236 [34.39]	1870 [52.03]	504 [14.02]	976 [27.16]	632 [17.58]	552 [15.36]	298 [8.29]	144 [4.01]	3106 [86.42]
^1^TN^2−1^		10 [0.28]		4 [0.11]	6 [0.17]				10 [0.28]
^1^TN^2−2^	2 [0.06]			2 [0.06]					2 [0.06]
^1^TN^3^		2 [0.06]		2 [0.06]					2 [0.06]
^2^TN^1^B^1−2^L^1^	2 [0.06]	2 [0.06]		2 [0.06]		2 [0.06]			4 [0.11]
^2^TN ^1^B^1^L^1^	64 [1.78]	78 [2.17]	34 [0.95]	56 [1.56]	24 [0.67]	22 [0.61]	2 [0.06]	4 [0.11]	142 [3.95]
^2^TN B^1^L^1^	16 [0.45]	2 [0.06]	2 [0.06]	4 [0.11]	4 [0.11]		6 [0.17]	2 [0.06]	18 [0.5]
^2^TN^1^B^1−2−1^L^1^	2 [0.06]	2 [0.06]		4 [0.11]					4 [0.11]
^3^TNMB^1^DB^1^L^1^	2 [0.06]						2 [0.06]		2 [0.06]
^3^TN^1^MB^1^DB^1^L^1^	2 [0.06]			2 [0.06]					2 [0.06]
C-shaped	8 [0.22]	2 [0.06]		6 [0.17]			4 [0.11]		10 [0.28]
Total	1480 [41.18]	2114 [58.82]	576 [16.03]	1164 [32.39]	722 [20.09]	638 [17.75]	338 [9.4]	156 [4.34]	3594 [100]

Additionally, the three-rooted, three-canal configuration (^3^TNMB^1^DB^1^L^1^, ^3^TN^1^MB^1^DB^1^L^1^) was identified in 0.11% of the examined premolars.

### Vertucci classification

According to the Vertucci classification, mandibular premolars most frequently exhibited Type I canals, followed by Type V canals (*p* < 0.001, *V* = 0.17). In particular, Type V canals were significantly more prevalent in mandibular first premolars than in second premolars. Conversely, Type V was observed significantly less frequently than expected in teeth #35 and #45 (Table 3).

**Table 3:** Distribution of premolar teeth according to Vertucci classification

	Left 1st PM	Right 1st PM	Left 2nd PM	Right 2nd PM	Overall
Vertucci classification	*n* [%]	*n* [%]	*n* [%]	*n* [%]	*n* [%]
Type I	714 [19.87]	726 [20.2]	844 [23.48]	826 [22.98]	3110 [86.53]
Type II	8 [0.22]	8 [0.22]		2 [0.06]	18 [0.5]
Type III	2 [0.06]		2 [0.06]	2 [0.06]	6 [0.17]
Type IV	12 [0.33]	6 [0.17]			18 [0.5]
Type V	192 [5.34]	178 [4.95]	28 [0.78]	22 [0.61]	420 [11.69]
Type VIII	2 [0.06]			2 [0.06]	4 [0.11]
Unclassified	4 [0.11]	4 [0.11]	4 [0.11]	6 [0.17]	18 [0.5]
Total	934 [25.99]	922 [25.65]	878 [24.43]	860 [23.93]	3594 [100]

**Fig. 2:**
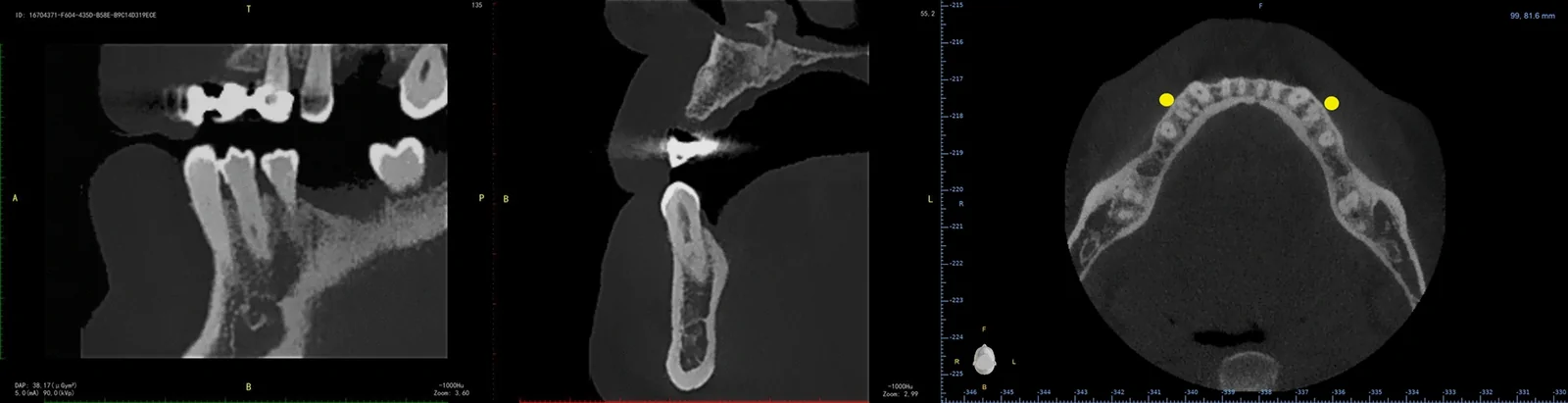
Bilateral cross-sectional and axial CBCT images for the ^3^TNMB^1^DB^1^L^1^ configuration

Bilateral assessment revealed no significant differences between Type I and Type V canals in the 34–44 and 35–45 tooth pairs (*p* = 0.281 and *p* = 0.327, respectively). The right–left agreement, expressed as kappa coefficients, was 0.624 for PM1 (34–44) and 0.463 for PM2 (35–45), indicating statistically significant symmetry in both cases (Fig. 2).

Analysis by sex revealed a statistically significant association between root canal configuration and sex (*p* < 0.001, *V* = 0.07). Type I was the most frequently observed configuration in both sexes, whereas Type V was observed more frequently than expected in males (OR = 1.54, 95% CI 1.25–1.90). No significant differences were found in the distribution of Vertucci Type I or Type V canals across age groups (*p* = 0.40, *V* = 0.05). The distribution of Vertucci class according to age group and sex is presented in Table 4.

**Table 4:** Distribution of Vertucci classification according to age group and sex

	Type I	Type II	Type III	Type IV	Type V	Type VIII	Unclassified	Overall
Age group	*n* [%]	*n* [%]	*n* [%]	*n* [%]	*n* [%]	*n* [%]	*n* [%]	*n* [%]
18–20	506 [14.08]				70 [1.95]			576 [16.03]
> 20–30	976 [27.16]	8 [0.22]	2 [0.06]	8 [0.22]	154 [4.28]	4 [0.11]	12 [0.33]	1164 [32.39]
> 30–40	632 [17.58]	10 [0.28]	4 [0.11]	6 [0.17]	70 [1.95]			722 [20.09]
> 40–50	552 [15.36]			2 [0.06]	82 [2.28]		2 [0.06]	638 [17.75]
> 50–60	300 [8.35]			2 [0.06]	32 [0.89]		4 [0.11]	338 [9.4]
> 60	144 [4.01]				12 [0.33]			156 [4.34]
Gender								
Male	1240 [34.5]			14 [0.39]	212 [5.9]	2 [0.06]	12 [0.33]	1480 [41.18]
Female	1870 [52.03]	18 [0.5]	6 [0.17]	4 [0.11]	208 [5.79]	2 [0.06]	6 [0.17]	2114 [58.82]
Total	3110 [86.53]	18 [0.5]	6 [0.17]	18 [0.5]	420 [11.69]	4 [0.11]	18 [0.5]	3594 [100]

**Fig. 3:**
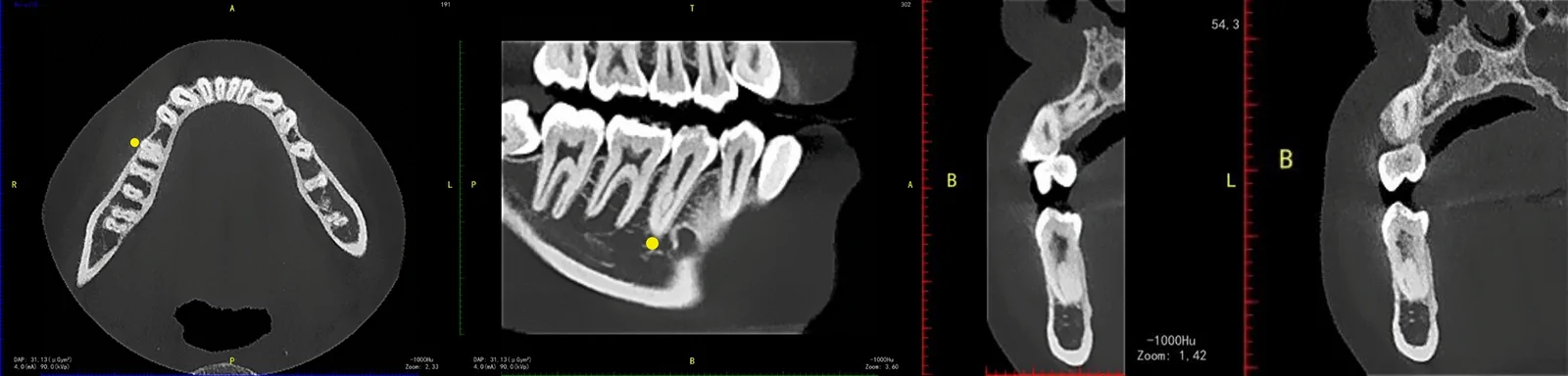
Axial, panoramic and cross-sectional CBCT scans showing the Vertucci Type III configuration

C-shaped canals were also evaluated separately, as this morphology is not included in either classification system. A C-shaped configuration (Fig. 3) was identified in 0.28% of the samples. Owing to its low frequency, no meaningful associations with age, sex, or tooth type were observed.

### Number of roots

Any root division occurring in the coronal, middle, or apical third was coded as 2 or more roots according to the new classification system. Accordingly, the prevalence of 2-rooted mandibular first premolars was significantly greater than that of second premolars (*p* < 0.001, *V* = 0.16).

### Assessment of the radicular groove

In the study sample, the initial position of the RG, defined as the linear distance measured along the root surface from the cementoenamel junction (CEJ) to the most coronal point where the groove became identifiable, was 5.09 ± 1.96 mm (min: 1.44 mm; max: 10.74 mm). The RG length, measured from this coronal starting point to its most apical extent, was 5.12 ± 1.75 mm (min: 2.00 mm; max: 11.93 mm). RG was observed significantly more frequently in mandibular first premolars (*p* < 0.001, *V* = 0.25).

**Fig. 4:**
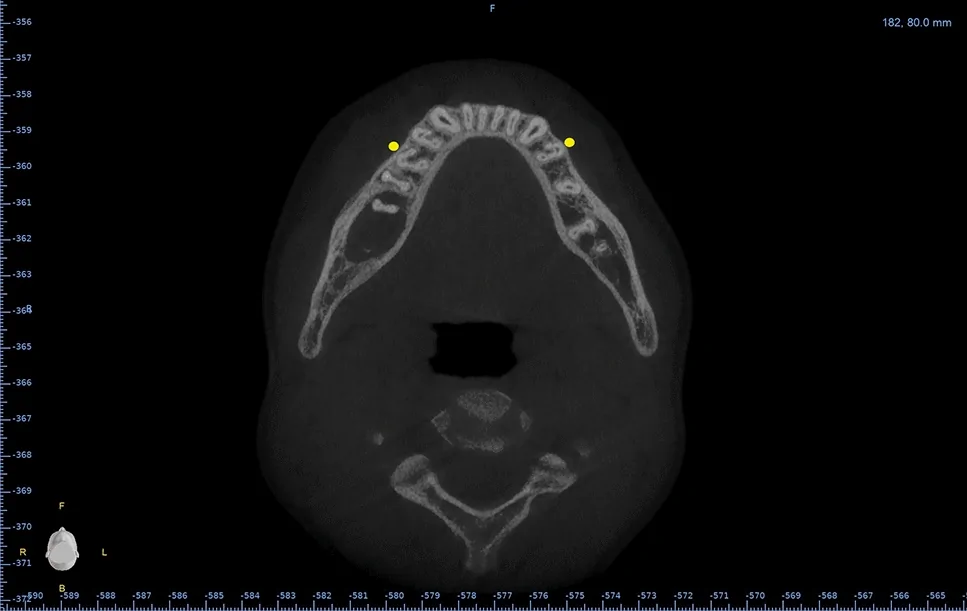
Axial cross-sectional images of the tooth at the cementoenamel junction (**a**), coronal third (**b**), middle third (**c**), and apical third (**d**). The radicular groove is clearly identifiable at the middle and apical thirds of the root

RGs were predominantly located on the mesial aspect of the roots, accounting for 86% of all the detected RGs (Fig. 4).

Bilateral symmetry of RG presence was assessed for first premolars. McNemar’s test indicated a statistically significant difference in RG presence between the right and left teeth (*p* < 0.01). However, Cohen’s kappa coefficient was 0.68, indicating good agreement between the two sides (*p* < 0.001). These results suggested that RG was generally symmetric in most individuals’ right and left first premolars, although in some cases, it was present only on one side.

The presence of RG according to the Vertucci classification was observed in 2% of Type I teeth (50/3110), 77.8% of Type IV teeth (14/18), 38.1% of Type V teeth (160/420), and 100% of Type VIII teeth (4/4).

## Discussion

The complexity of the root canal system has long been investigated in numerous experimental and clinical studies, which have demonstrated that root and canal morphology can vary significantly between populations, within the same population, and even within the same individual [7, 8, 18]. In this context, CBCT has become one of the most reliable methods for detailed three-dimensional assessment of root canal anatomy [19]. Compared with conventional 2-dimensional imaging, CBCT provides high-resolution 3-dimensional images of small areas with lower radiation exposure and allows accurate evaluation not only of root canal morphology, but also of complications such as instrument fracture, root perforation, fractures, and canal calcifications [19].

This study aims to leverage the high-resolution imaging capabilities of CBCT to comparatively evaluate the root canal morphology of mandibular first and second premolars (M1PMs and M2PMs) using both the conventional Vertucci classification and the new root canal classification system proposed by Ahmed et al. [8, 20]. Accordingly, this study seeks to identify morphological variations in mandibular premolars in a Turkish subpopulation and to determine the additional clinical and morphological insights provided by the new system compared with the traditional classification system.

To reduce the complexity arising from the diversity of canal morphology and to more accurately define canal configurations, various classification systems have been proposed in the literature. The most commonly used systems were developed by Vertucci et al. [8] and Weine, and these systems have long served as fundamental references for describing root canal configurations. However, recent studies using advanced imaging technologies have shown that the root canal morphology is more complex than previously expected and that some canal configurations cannot be fully explained by the existing classification systems [21, 22]. In fact, Filpo-Perez et al. [23] reported that 13% of their samples did not conform to the Vertucci classification.

Existing classification systems have certain limitations. For example, in the widely used Vertucci classification, 2-rooted mandibular premolars are not assigned to a separate category, whereas the mesial and distal roots of lower molars are classified independently. Similarly, Vertucci et al. designated 3-canal maxillary premolars as Type VIII, but no details regarding the number of roots were provided. Clinically, knowledge of the number of roots is as important as the number of canals, as it directly affects access cavity design, the direction of mechanical preparation, and the efficacy of canal obturation procedures [24]. These findings indicate that existing systems may be insufficient to fully represent the anatomical diversity of certain tooth groups. In the present study, 5% of the examined samples did not conform to the Vertucci classification.

While Vertucci’s system identifies 3 canal configurations, it does not provide information on root number. Ahmed’s classification offers a more detailed framework by allowing simultaneous identification of canal and root morphology, facilitating the detection of the 3-rooted premolars in this study. However, despite this advantage, descriptions of the precise level at which root division occurs remain limited.

**Fig. 5:**
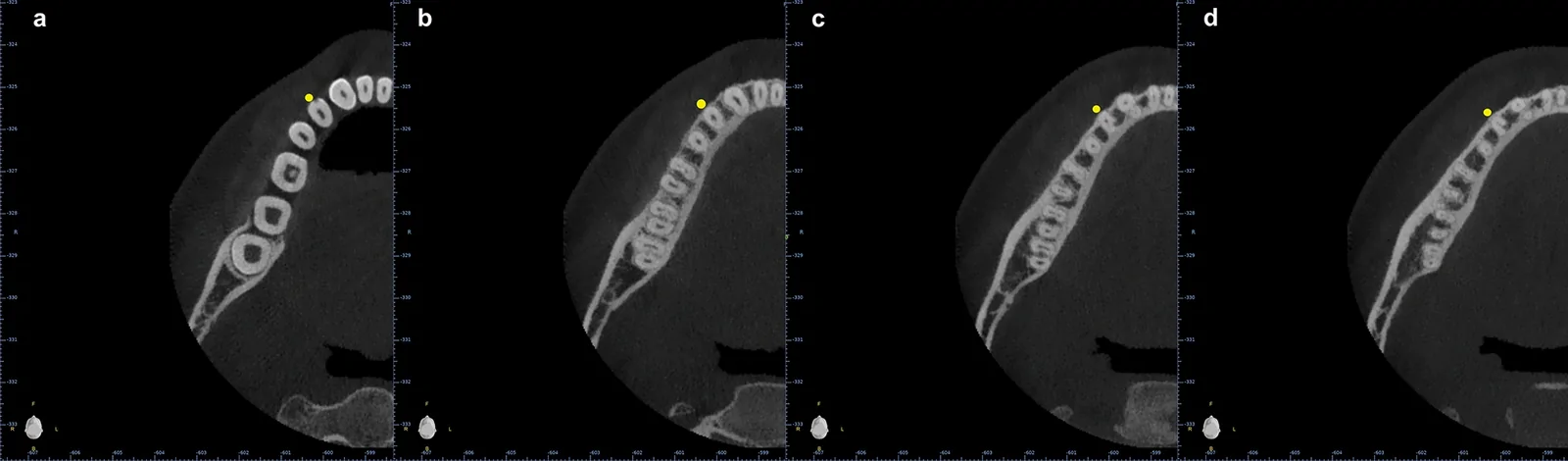
Axial cross-sectional images of the tooth at the cementoenamel junction (a), coronal third (b), middle third (c) and apical third (d). The radicular groove is clearly identifiable at the middle and apical thirds of the root

A tooth appearing as Vertucci Type III on panoramic radiography is shown in Fig. 5, but axial CBCT sections revealed two roots and 3 canals. This observation underscores the importance of CBCT in precise anatomical assessment and the added descriptive value of Ahmed’s classification.

Various root canal configurations have been reported in the literature, including 4-rooted maxillary molars, 3-canal premolars, and mandibular molars with a distolingual root [24–26]. These findings indicate that root canal systems can exhibit significant variations depending on tooth type and individual morphology.

Despite the contributions of various researchers, these studies demonstrate that a simple, universal classification system encompassing all tooth types and canal morphologies has not yet been established. To address these limitations, a new classification system was developed and utilized in the present study. The proposed system does not include parameters such as root and canal curvature, exact bifurcation levels, root fusion types, lateral and furcation canals, or apical deltas. While the inclusion of these parameters could theoretically provide a more detailed and anatomically accurate system, it may also complicate the classification and lead to interpretative discrepancies [24]. During the development process, incorporating such details significantly increased complexity and had the potential to generate variability in interpretation.

Ahmed et al. [20] in a subsequent update, proposed the integration of accessory canals and other morphological variations into the classification system. This update aimed to expand the anatomical scope of the previous system, particularly emphasizing the potential impact of lateral and furcation canals on endodontic treatment outcomes. However, incorporating these additional features could increase the complexity of the classification and potentially reduce its practicality in clinical applications.

The evaluation of apical canal configurations can vary depending on the method used (experimental or clinical), which may lead to interpretative differences among observers. In some studies, apical bifurcations, apical deltas, or minor divisions branching from the main canal (Types I–II) have been reported. Therefore, the assessment of apical configurations should consider the specific methods and criteria applied. In the present study, root canal configuration data were recorded following the classification approach proposed in the referenced article.

The new system excludes developmental anomalies and minor canal anatomies. Additionally, interpretation can be challenging for some 2-rooted teeth. For example, when bifurcation occurs in the coronal third, it is expressed as ^2^14B^1^P^1^, whereas bifurcation in the middle or apical third is expressed as ^2^14^1^B^1^P^1^. Although this coding does not precisely indicate the exact bifurcation location, it provides meaningful information regarding the root canal configuration.

In the present study, the most common root canal configuration was ^1^TN^1^, followed by ^1^TN^1–2^. These findings are consistent with those of previous studies in the Turkish population [27]. Similarly, the predominance of single-rooted mandibular premolars has been widely reported in the literature, with studies showing a single-root frequency of approximately 95–97% and a low prevalence of 2 or 3 roots [1]. In other populations, however, the ^1^TN^1^ configuration is often followed by 2TNB^1^L^1^, indicating that root canal morphology may vary according to ethnic and geographic factors [28].

Previous studies have also reported a relatively high prevalence of multiple canals and C-shaped morphology, particularly in mandibular first premolars. In contrast, C-shaped canal morphology was identified in only 5 premolars in the present study, which is consistent with earlier reports indicating a frequency of 0.24–0.40% in comparable populations [29]. This lower frequency may be related to population-specific anatomical characteristics and differences in sample composition.

In addition, while one study conducted in the Saudi subpopulation reported a significant association between MPM root canal morphology and age, another CBCT-based study revealed no significant differences according to age or sex and reported ^1^TN^1^ as the predominant configuration [30]. Similarly, a study conducted in the Pakistani population reported a predominance of single-rooted premolars, with no significant differences according to age or sex [31]. In the present study, no relationship between age and canal configuration was observed in the Turkish subpopulation. However, a significant association was found between canal configuration and sex, with Vertucci Type V being more frequently observed in males. Taken together, these findings suggest that root canal morphology may vary according to population characteristics, sample composition, and demographic factors [1].

Our findings indicate that the new classification system is more capable of capturing age-related variations in root canal morphology than the traditional Vertucci classification, highlighting its superiority in reflecting clinically relevant anatomical nuances.

In the current study, the presence and morphological characteristics of RGs in mandibular premolars were also evaluated. RGs are typically located on the mesial and mesiobuccal surfaces and may be associated with accessory canals (RGAC) [9]. Clinically, RGs can serve as bacterial reservoirs, contributing to the development of localized periodontitis and potentially adversely affecting treatment prognosis. In this study, RGs were observed significantly more frequently in mandibular first premolars and were particularly concentrated in the middle third of the root in Vertucci Type V and in the ^1^TN^1–2^ and ^2^TNB^1^L^1^ configurations according to Ahmed’s classification. Previous studies have reported that RGs often originate on the mesial surface and may be associated with C-shaped or multiple canal configurations according to cross-sectional root analyses [9]. Similarly, in the present study, RGs were predominantly located on the mesial and mesiobuccal surfaces and were associated with increased complexity of the root canal morphology. Consistent with these findings, a micro-CT study conducted on teeth with deep radicular grooves demonstrated a high prevalence of complex canal systems, with multiple canals present in almost all samples and a wide range of root canal configurations identified [32]. Consistent with these findings, other population-based studies have reported a higher prevalence of anatomical complexities, including multiple canals and radicular grooves, particularly in mandibular first premolars. Although differences in prevalence rates have been reported among populations, these variations may be attributed to ethnic factors, sample characteristics, and differences in the classification systems used to describe root canal morphology [28].

In mandibular premolars with RGs, radicular groove-associated accessory canals (RGACs) are frequently observed and are typically located in the middle third of the root. This structure can contribute to the deepening of periodontal pockets and the appearance of clinical findings that may be mistaken for vertical root fractures. Moreover, the incidence of RGACs has been reported to increase with increasing internal and external morphological complexity of the root [9, 32, 33]. Consistent with these reports, the present study revealed that RGs were significantly more prevalent in the middle third of M1PMs. Therefore, the presence of RGs and RGACs in M1PMs highlights the need for clinicians to ensure adequate shaping of the main canals and effective irrigation during endodontic treatment. Nevertheless, despite detailed morphological characterization, clinical access to RGACs remains challenging. In mandibular premolars with RGs, radicular groove-associated accessory canals (RGACs) are frequently observed and are typically located in the middle third of the root. This can contribute to the deepening of periodontal pockets and the appearance of clinical findings that may be mistaken for vertical root fractures. Moreover, the incidence of RGACs has been reported to increase with increasing internal and external morphological complexity of the root [9, 32, 33]. Consistent with these reports, the present study revealed that RGs were significantly more prevalent in the middle third of M1PMs. Therefore, the presence of RGs and RGACs in M1PMs highlights the need for clinicians to ensure adequate shaping of the main canals and effective irrigation during endodontic treatment. Nevertheless, despite detailed morphological characterization, clinical access to RGACs remains challenging.

RGs were observed in 2% of teeth with Vertucci Type I configurations, 77.8% of those with Type IV, 38.1% of those with Type V, and 100% of those with Type VIII teeth. These findings are consistent with the literature, indicating a positive correlation between increasing complexity of root canal morphology and the prevalence of RGs [33]. Similarly, a study conducted in a Chilean population revealed that mandibular first premolars with multiple root canals exhibited a higher incidence of radicular grooves, which were closely associated with more complex internal anatomy [34]. Additionally, the distance from the cementoenamel junction (CEJ) to the RG origin and the mean RG length were measured, emphasizing that the presence of RGs and RGACs should be carefully considered during endodontic treatment of mandibular premolars [9, 33].

In addition to evaluating RGs, the present study also performed an analysis of the FCAP, which identifies the region where 2 canals become distinctly separate. The findings indicated that in MPMs, particularly in Vertucci Type V canal configurations, the FCAP was most frequently located in the middle third of the root, followed by the apical third. Similarly, according to the new classification, the FCAP was predominantly located in the middle third of the root for the ^1^TN^1−2^ and ^2^TNB^1^L^1^ configurations and less frequently in the apical third. In line with these findings, a micro-CT study conducted in a Chilean population reported a high incidence of isthmuses, most of which were located in the middle third of the root canal, indicating that anatomical complexities tend to concentrate in this region [34].

Previous studies have primarily examined the FCAP in maxillary first molars and reported that it is largely situated in the coronal third of the root [35]. However, no studies to date have evaluated the distribution of FCAP in mandibular premolars. Therefore, the present findings provide some of the first data on the level of canal separation in MPMs, demonstrating that the FCAP is most commonly located in the middle third of the root. These results suggest that variations in root canal morphology and canal separation levels in MPMs may differ substantially from those observed in maxillary molars.

## Conclusion

In this study, the root canal morphology of mandibular premolars in a Turkish subpopulation was evaluated using CBCT and Ahmed’s new classification system. No significant differences were observed among the age groups, but bilateral morphological symmetry was noted. The Vertucci Type V configuration was more frequently observed in males. Radicular grooves were predominantly located in the middle third of the root and were associated with the canal morphology. The 3-dimensional evaluation provided by CBCT significantly contributed to the characterization of the complex anatomy of mandibular premolars and supported the clinical applicability of the new classification system.

## Limitations

This study has several limitations. Given that the research was conducted at a single center, the sample size was limited, which may restrict the generalizability of the findings. Additionally, the CBCT images were evaluated retrospectively, which could influence the results. Although the CBCT images were acquired with a voxel size of 0.3 mm, which is commonly used in clinical practice, this resolution may be insufficient to capture very fine anatomical details compared with smaller voxel sizes (≤ 0.2 mm) or micro-CT and nano-CT imaging. Therefore, subtle anatomical structures might not have been fully detected.

To more accurately determine the morphological distribution of mandibular premolars in the Turkish population, future studies with larger sample sizes, multicenter designs, and standardized imaging protocols are recommended.

## Data Availability

The datasets used and analyzed during the current study are available upon reasonable request.
